# Atomic Force Microscopy: A Versatile Tool in Cancer Research

**DOI:** 10.3390/cancers17050858

**Published:** 2025-03-02

**Authors:** Francesca Persano, Alessandro Parodi, Tatiana Pallaeva, Ekaterina Kolesova, Andrey A. Zamyatnin, Vadim S. Pokrovsky, Valeria De Matteis, Stefano Leporatti, Mariafrancesca Cascione

**Affiliations:** 1 Mathematics and Physics Department “Ennio De Giorgi”, University of Salento, Via Arnesano, 73100 Lecce, Italy; francesca.persano@unisalento.it (F.P.); valeria.dematteis@unisalento.it (V.D.M.); 2 CNR Nanotec-Istituto di Nanotecnologia, Via Monteroni, 73100 Lecce, Italy; 3 Scientific Center for Translation Medicine, Sirius University of Science and Technology, 354340 Sochi, Russia; parodi.a@talantiuspeh.ru (A.P.); tatiana.pallaeva@gmail.com (T.P.); kolesova.ep@talantiuspeh.ru (E.K.); vadimpokrovsky@yandex.ru (V.S.P.); 4 Federal Scientific Research Center Crystallography and Photonics, Russian Academy of Sciences, 119333 Moscow, Russia; 5 Department of Biological Chemistry, Sechenov First Moscow State Medical University (Sechenov University), 119991 Moscow, Russia; zamyat@belozersky.msu.ru; 6 Belozersky Institute of Physico-Chemical Biology, Lomonosov Moscow State University, 119992 Moscow, Russia; 7 Faculty of Bioengineering and Bioinformatics, Lomonosov Moscow State University, 119234 Moscow, Russia; 8 N.N. Blokhin Medical Research Center of Oncology, 115478 Moscow, Russia; 9 Patrice Lumumba People’s Friendship University, 117198 Moscow, Russia; 10 Institute for Microelectronics and Microsystems (IMM), National Research Council (CNR), Via Monteroni, 73100 Lecce, Italy

**Keywords:** atomic force microscopy, cancer cell, epithelial to mesenchymal transition, cytomechanics

## Abstract

The implementation of novel analytic methodologies in cancer and biomedical research has enabled the quantification of parameters that were previously disregarded only a few decades ago. A notable example of this paradigm shift is the widespread integration of atomic force microscopy (AFM) into biomedical laboratories, significantly advancing our understanding of cancer cell biology and treatment response. AFM allows for the meticulous monitoring of different parameters at the molecular and nanoscale levels, encompassing critical aspects such as cell morphology, roughness, adhesion, stiffness, and elasticity. These parameters can be systematically investigated in correlation with specific cell treatment, providing important insights into morpho-mechanical properties during normal and treated conditions. The resolution of this system holds the potential for its systematic adoption in clinics; its application could produce useful diagnostic information regarding the aggressiveness of cancer and the efficacy of treatment. This review endeavors to analyze the current literature, underscoring the pivotal role of AFM in biomedical research, especially in cancer cases, while also contemplating its prospective application in a clinical context.

## Simple Summary

The integration of atomic force microscopy (AFM) into biological facilities, is significantly increasing our understanding of tumors biology and answer to treatments. AFM allows for investigating cell morphology, roughness, adhesion, stiffness, and elasticity in correlation with specific cell treatment, by comparing the morpho-mechanical properties in healthy and treated cells, thereby allowing diagnostic information of the aggressiveness of cancer and the efficacy of any treatment. This review attempts to analyze current literature, highlighting the role of AFM in biomedical research and specifically in cancer case, while also shading some light into novel possible applications in clinics.

## 1. Introduction

For many years, the relationship between biomechanics and medicine has been deeply investigated in the scientific community in order to establish a relationship between biological function, cellular structure, mechanical properties, and health/disease states [1,2]. In detail, mechanobiology is currently one of the new fields of basic research [3]. It initially focuses on understanding the role that physical forces play in cellular communication and the regulation of functions and, subsequently, delves into the involvement of these forces in more complex processes such as tissue and organ development, ultimately encompassing the origin and progression of diseases [4,5], including cancer.

Cancer, often a fatal disease, owes its lethality primarily to its invasive and metastatic potential, which can be linked to mechanotype alterations in individual tumor cells and changes in the cancer microenvironment. This invasive capability frequently serves as the threshold distinguishing precancerous lesions from cancerous ones.

Although numerous studies have been conducted to understand the potential biomolecular pathways involved in cancer progression [6], their comprehension remains not entirely clear, as they are highly variable and interconnected. In particular, the molecular mechanisms underlying the regulation of cellular mechanotypes are intricate and not fully examined, although it is widely accepted that they involve complex biological processes such as pathways associated with cell adhesion [7], cytoskeletal [8] and nucleoskeletal [9] remodeling, and epithelial-mesenchymal transition (EMT) [10].

In recent decades, various methodologies and techniques have been applied to quantify these cellular mechanotype changes in tumors. Compelling evidence has emerged indicating that cellular mechanotype changes, such as alterations in cellular rigidity and deformability, are common phenotypic events in the onset and progression of cancer [11,12].

Among various techniques, AFM stands out as the predominant tool for characterizing both the mechanical and morphological aspects of cells, even in live conditions. This proficiency has established AFM as a widely employed instrument for the comprehensive morphomechanical characterization of cells.

## 2. Concise Overview of AFM

### 2.1. Principles of Operation

AFM belongs to the family of scanning probe microscopes (SPMs), so named to indicate that the observation of the sample surface is obtained through the pointwise interaction between the atomic layers of the investigated sample and a physical probe that scans them. AFM stands as the pre-eminent member of the SFM family, renowned for its unparalleled resolution. It boasts a horizontal resolution of 0.1 nm and a vertical resolution of 0.01 nm. In addition, measurements can be conducted in air or in liquid conditions, solidifying its status as the most extensively employed instrument in its class.

In the case of AFM, the probe consists of a nanometric tip (~a few nm) fixed at the free end of a flexible microcantilever. The movement of the tip over the sample is achieved by two ceramic piezoelectric systems that allow the movement of the probe relative to the sample in the *xy* plane and in the *z* direction. During scanning, the height differences of the sample punctually modify (with sub-nanometric resolution) the relative distance between the sample and the tip’s end, leading to a change in the interaction force (≥pN) that induces a deflection of the cantilever. This deflection is extremely small regarding detection; therefore, typically, cantilever deflection is tracked by reflecting a laser beam onto the back of the cantilever, directing it to a four-quadrant photodetector, allowing quantification of both bending and torsional movements, respectively, due to normal forces (attraction/repulsion according to Lennard-Jones potential) and lateral forces (friction).

### 2.2. Topographic Techniques

AFM provides a diverse array of measurement modes that empower researchers to characterize various properties depending on the specific sample intended for characterization. These modes span from high-resolution surface topography to the assessment of electrical or magnetic properties.

In this review, we will confine ourselves to describing the most used imaging techniques for the characterization of biological samples, including cancer cells. These methods encompass (i) a contact mode (operating at constant force) and (ii) a tapping or vibration mode.

The contact mode represents the original and most straightforward method of operation. In this mode, the probe remains in continuous contact (at a relative distance, ~Å) with the sample while raster scanning its surface. The prevalent configuration of the static mode often involves operating it in constant force or deflection feedback mode, in which the cantilever deflection serves as the feedback parameter, with the user setting the deflection, determining the force exerted by the tip against the surface. This allows the user to control the magnitude of the interaction between the probe and the sample.

In detail, the vertical (z) position of the sample (or tip) is monitored by an electronic feedback circuit, maintaining a constant interaction force (i.e., cantilever deflection) between the cantilever tip and sample. The required z variation to sustain a constant interaction force is graphed against the x and y coordinates, generating a topographic image. This signal is then transmitted to an electronic feedback circuit, which generates the necessary voltage for the piezo to maintain a constant tip–specimen distance. Consequently, the system follows the sample surface contour, producing a topographical image. However, this technique has limitations regarding biological samples due to their softness; therefore, AFM imaging has to be performed gently by adjusting the tip–sample interaction force at a minimum upon acting on scanning parameters like setpoint voltage. Therefore, in order to measure softer samples, AFM Tapping Mode and other even more advanced techniques (such as Peak Force Tapping Mode) were later developed.

In the tapping or vibration mode, a piezoelectric element on the tip holder induces the cantilever to oscillate near its resonance frequency.

The cantilever is subsequently subjected to sinusoidal motion with a constant excitation energy akin to a damped spring or a single harmonic oscillator. When the oscillating cantilever scans the sample surface, local changes in amplitude occur due to alterations in the cantilever’s operational frequency and, hence, changes in the measured amplitude. The latter is detected using a two- or four-quadrant photodiode, and it is converted into an electrical signal, which serves the electronic feedback system to apply the appropriate voltage to the piezo; this restores the amplitude (or frequency) to that corresponding to the normal resonant condition of the probe. The feedback loop signal is recorded and analyzed to digitally reconstruct the morphology of the investigated sample.

### 2.3. Static and Dynamic Force Spectroscopy Characterization

AFM force spectroscopy is a technique designed to quantify the local mechanical properties of the surface of a sample. It is based on the analysis of indentation curves, which was introduced at the beginning of the 1970s and was then optimized by technological development; nowadays, it is a conventional methodology used to estimate materials’ elastic properties at micro- and nanometric levels at high resolution.

**Figure 1:**
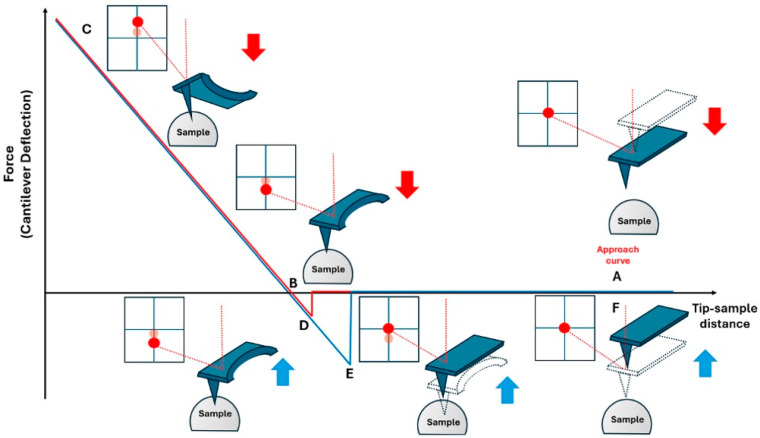
Force (cantilever deflection) versus tip–sample distance curve cycle and corresponding photodetector signal changes. (A) AFM cantilever is far from sample: no interaction between tip and sample. (B) AFM is approaching the sample, cantilever is deflected towards the sample. (C) AFM cantilever is in contact with the sample. (D) AFM cantilever adheres into the sample (E) AFM tip starts to retract from the sample (F) AFM tip is fully retracted from the sample.

The indentation curves represent the interaction force between the tip and the sample and are related to their relative distance; it is composed of two curves corresponding to the approach and retraction of the probe with respect to the surface sample (see Figure 1).

In detail, to generate the FD curve, the cantilever and the sample are initially distanced so as not to interact (A); subsequently, the tip (or the sample) is incrementally moved along the vertical axis (*z*), reducing the relative tip–sample distance, therefore the cantilever perceives the long-range attractive force, *F* (mainly due to electrostatic forces first, and then van der Waals forces) that deflect the cantilever downward (B-C). The cantilever deflection (δ_c_), revealed through the variation in vertical signal onto the photodiode, converts the electronic signal into nanometric tip deflection (this requires the estimation of a sensitivity parameter). By measuring δ_c_ and knowing *k_c_*, it is possible to quantify the interaction force *F* using Hooke’s law. As the distance between the tip and the sample diminishes, the gradient of the interaction force increases until it surpasses the cantilever force constant, leading to the tip abruptly contacting the surface (contact point). From this point onward, the tip induces a surface sample deformation, and the cantilever undergoes a positive deflection until the maximum value (trigger) is reached (fixed by the operator). Subsequently, the feedback system begins the process of retracting the tip from the surface of the sample; the cantilever bends towards the surface owing to the adhesion force until the cantilever force constant surpasses the gradient of the adhesive interaction force; the tip loses contact and returns to the starting point. Specifically, when the probe approaches the sample (Figure 1), it feels attraction toward the surface due to predominantly capillary, van der Waals, and electrostatic forces. At point B, these negative forces overcome the stiffness of the cantilever, allowing the tip to be drawn to the surface and, furthermore, initiating indentation into the sample until the z position of the modulation arrives at its maximum (point C). This position means that the peak force value is used for feedback control. After this point, the probe starts to withdraw until it reaches the pull-off point (the maximum adhesion point, which is the minimum force). The tip then retracts further and returns to its original position (E), where, akin to point A, no external force field influences its motion.

In summary, the FD curve is composed of an approach and forward data, which gives information about the elastic properties of the sample and adhesion, respectively.

A single indentation measurement does not provide topographical information, which is essential for correlating the mechanical response to specific points on biological samples, as their surfaces are inherently heterogeneous.

**Figure 2:**
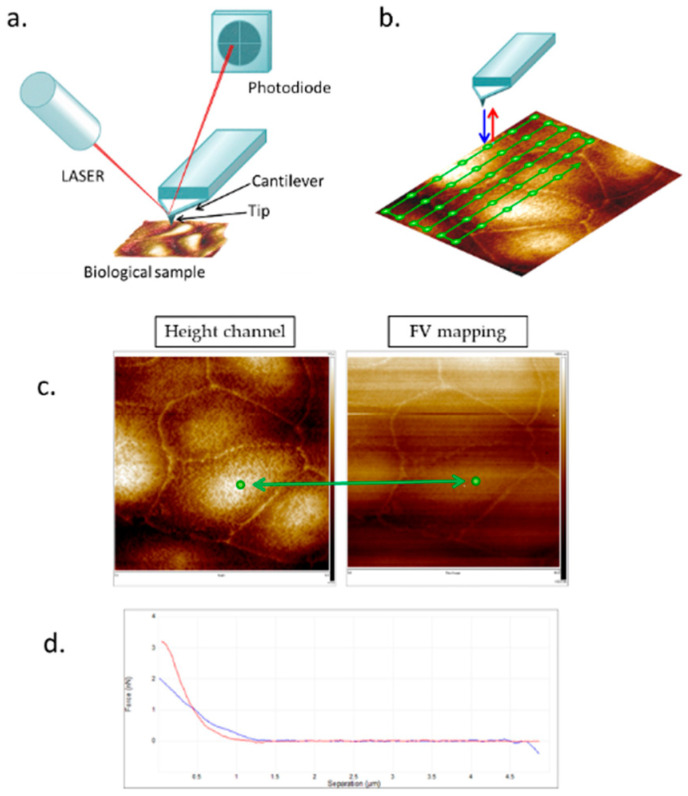
Scheme of the force-volume working mode. In a is drawn a typical (Bio)AFM set up. The green line (indicated in (**b**)) shows the raster motion of the AFM probe on the sample surface. At the points marked, the tip performs a complete run of indentation (up and down as indicated by red and blue arrows). Images (**c**–**d**) represent a screenshot of the interface acquisition and analysis software; topography (Height channel) and the force-volume channel (FV channel) are simultaneously acquired. The pixels of the two images are closely connected; every pixel of the canal’s topography (in (**c**)) is directly correlated to a force–distance curve (shown in (**d**) and indicated with red line (approach) and blue (retracted curve)). Reproduced with Permission from Cascione et al. [14].

To overcome these limits of the force mode, force-volume (FV) imaging was introduced. When operating in contact mode, the tip performs topography over a selected area of the sample (see Figure 2). At each pixel, the probe conducts a nanoindentation, allowing for the mapping of the mechanical response of the sample surface [13,14].

A similar analysis can be conducted when operating in dynamic mode.

The distinction between the static and dynamic modes of AFM operation lies in the measurement of interaction forces. In static-deflection AFM, the interaction force between the tip and the sample is directly obtained using Hooke’s law. Conversely, in dynamic mode, changes in the resonant frequency, amplitude, and phase of oscillation occur due to interaction forces and dissipative processes.

The acquisition of FD curves is physically similar to static mode acquisitions, with the difference being that the movement of the tip is sinusoidal rather than triangular. When the oscillating tip is far from the surface, it is not affected by any force. As it approaches the surface, the cantilever is deflected toward it due to attractive forces (typically van der Waals, electrostatic, or capillary forces). The tip remains on the surface, and the force increases until the z position of the modulation reaches its lowest level, in which the peak force occurs. The peak force during the interaction period is kept constant by the system’s feedback. Subsequently, the probe begins to retract, and the force decreases until it reaches a minimum, at which point adhesion is measured. The point at which the tip detaches from the surface is called the pull-off point, which often coincides with the minimum force. Once the tip has detached from the surface, only long-range forces affect the tip, so again, the force is very small or negligible when tip–sample separation is at its maximum.

The cantilever is oscillated at a specific frequency by applying a modulated voltage to the piezoelectric scanner. During indentation, the amplitude and phase shift of the cantilever’s deflection signal are detected using a lock-in amplification program and used to estimate the storage modulus (G′) and the loss modulus (G″) as functions of frequency and the relative tip–sample distance. Frequency scanning allows for an unequivocal separation of the elastic and viscous response, thus obtaining the storage and loss modulus as a function of frequency.

## 3. AFM Integrated with Optical Technique

**Figure 3:**
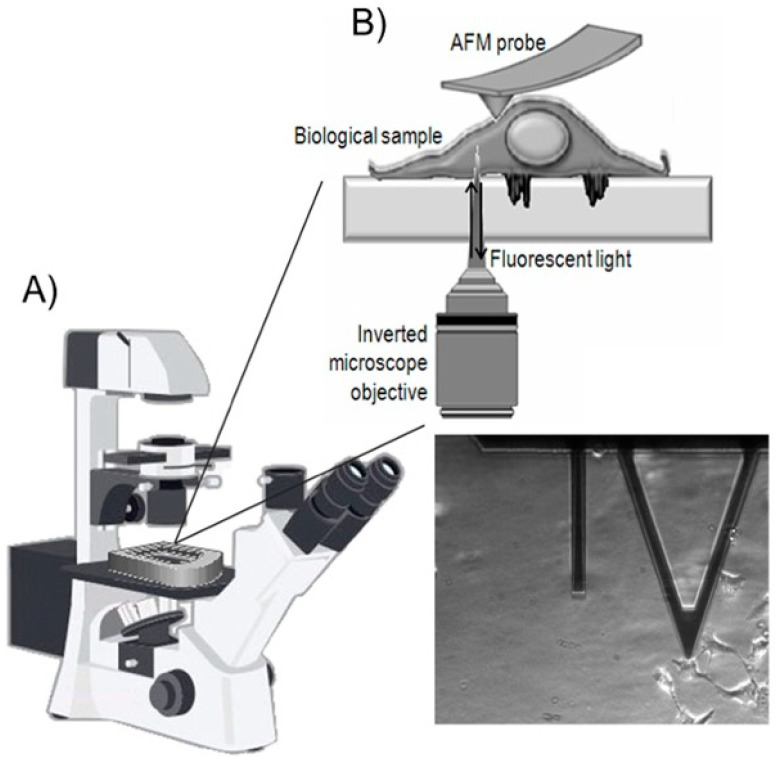
(**A**,**B**) Representation of AFM, as seen on an inverted optical microscope and optical image (20× objective). Positioning of the AFM tip on the surface of one cell. Adapted with Permission from Cascione et al. [16].

The comprehension of the profound inter-relation between biochemical pathways and cyto-mechanical response inevitably requires a comprehensive investigation into the mechanical properties contingent upon their physiological states. In this perspective, the data acquired using AFM must be compared with information derived from other techniques, primarily optical and fluorescence microscopies. In this perspective, numerous studies have been conducted concurrently. However, in some instances, correlating the results obtained using different techniques can be a hurdle due to the intrinsic heterogeneity of the investigated biological sample. To overcome this issue, hybrid systems have been developed, enabling the visualization of the sample using both an optical system and AFM [15]. Specifically, by employing an inverted microscope configuration, it is possible to view the sample from below, exploiting the transparency of biological samples, and map its surface from above using an AFM probe. Therefore, a hybrid setup is utilized to simultaneously record AFM and transmitted light, differential interference contrast microscopy (DIC), and phase contrast microscopy (PCM) acquisitions. From a practical standpoint, this coupling facilitates the positioning of the AFM probe on the chosen sample (Figure 3 [16]).

Moreover, the implementation of specific components in the instrumental setup allows for operation in different working configurations, especially fluorescence, which enables the simultaneous assessment of specific biological processes in terms of mechanical and morphological variations in the specific structural elements of a cell.

Specifically, in order to investigate the biological event in the contact region of the cell and substrate, total internal reflection fluorescence (TIRF) is broadly used; therefore, the implementation of a hybrid system with AFM showed the transmission forces within the cell from the apical to the basal membranes, monitoring focal contact dynamics in cultured cell monolayers [17].

**Figure 4:**
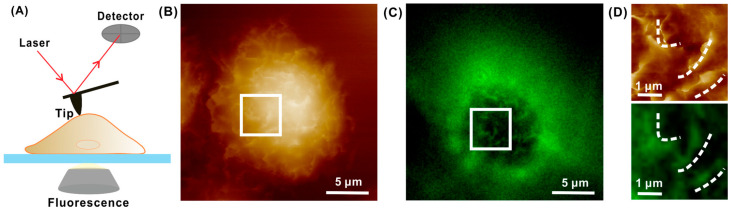
Schematic presentation of coupling AFM with fluorescence modulus (**A**). Correlative AFM (**B**) with CLSM (**C**) images of the ACFs on the surface of an HCT-116 cell stained with Fluo5 488 Phalloidin. Zoom (**D**) of the same region of the cell in the AFM (**upper**) and CLSM images (**bottom**) (correspondent to the white squares in (**B**,**C**), respectively). Reproduced with Permission from Liu et al. [20].

The tracking of dynamic processes within membranes is achievable through the utilization of membrane-targeted probes and CCD cameras in epifluorescence microscopy. Numerous instances of combined AFM and fluorescence microscope setups have been documented in the literature over the past few decades [18]. While effective for certain membrane model systems, this approach encounters challenges in numerous cases due to undesirable out-of-focus fluorescence, thereby diminishing the signal-to-noise ratio and impeding image resolution. These limitations are overcome using advanced microscopy techniques, offering significant enhancements for the in-depth study of dynamic membrane processes. Specifically, confocal microscopy surpasses conventional (widefield) microscopy by offering improved optical sectioning; consequently, it assumes a crucial role in providing detailed structural information about the biological specimen (Figure 4). The integrated CLSM+AFM system offers significant advantages, not only in aligning topographical information from AFM with high-resolution fluorescence images but also in facilitating the association of the mechanical response, recorded by AFM, with the visualization of the dynamic rearrangement throughout the entire cell volume of specific structural components [19,20].

Specialized live-cell fluorescence techniques encompass FRAP (Fluorescence Recovery After Photobleaching), which stands as a microscopy technique widely incorporated into commercial confocal microscopes, offering valuable insights into the dynamic attributes of fluorophores within the sample. The outcomes, contingent upon the intricacy of the data analysis, have the capacity to either qualitatively depict alterations in sample dynamics or generate quantitative data pertaining to the diffusion coefficients and membrane binding constants of the studied molecules [21]. Skamrahl et al. [21] devised and implemented an innovative optomechanical platform that integrates AFM and FRAP, which enables the concurrent measurement of cell mechanics and the associated actin kinetics; this allows for the quantification of the adaptive mechanical behavior of living cells.

The combination of an AFM with a CLSM capable of measuring the fluorescence lifetimes of the dyes represents another promising hybrid system in the field of cellular research. FLIM leverages the inherent properties of fluorescent dyes. Beyond possessing distinct emission spectra, each fluorescent molecule exhibits a characteristic lifetime, indicating the duration it remains in the excited state before emitting a photon. This lifetime serves as a distinctive parameter for each fluorescent dye or molecule, susceptible to alterations based on the nanoscopic environment (e.g., ion concentration, pH, and lipophilicity) or its conformational state (interaction with other molecules) [22]. FLIM seamlessly integrates lifetime measurements with imaging; the obtained lifetimes at the pixel level are color-coded to generate images. FLIM not only reveals the spatial distribution of a fluorescent molecule but also provides insights into its nano-environmental characteristics. Therefore, the combination of FLIM with AFM potentially yields information about biological processes occurring inside cells. However, only Becerra and colleagues [23] use the AFM+FLIM system to perform cellular investigation; they monitor the intracellular calcium changes of cardiac myocytes and the changes in the elastic modulus while applying controlled substrate deformations.

In contrast to confocal microscopy, stimulated emission depletion (STED) microscopy operates without being constrained by the inherent limitations of diffraction. A typical STED microscope utilizes a pair of co-aligned scanning-focused laser beams. One of these beams is dedicated to excitation, while the other assumes a donut-shaped configuration specifically designed for STED to prevent the de-excitation of potentially excited molecules through stimulated emission. Through the fluorescent labeling of specific proteins, it becomes feasible to track the processes and dynamics of these components within live cells in real time.

## 4. Current Uses and Importance of AFM in Cancer Biomedicine

Mechanobiology represents an emerging field of biophysical research; its goal is to unravel the complex interactions between mechanical forces and biological processes, shedding light on how these forces impact cellular function, contributing to health and disease conditions [3]. Cells are living mechanical entities that maintain a constant dynamic equilibrium of forces. Internal forces, generated by the pulling action of actin fibers within the cytoskeleton, are counterbalanced by resisting forces in the extracellular matrix (ECM). Additionally, cells are continuously exposed to external mechanical stimuli, including shear, compression, and tension forces. In response to these forces, cells adjust their shape, internal organization, and behavior through the activation of biochemical pathways. The latter triggers downstream responses, leading to changes in the activity of the transmembrane proteins and regulation of specific gene expression. Cellular morphology and mechanical behavior are strictly related to intracellular biological processes and, eventually, to human health conditions. Therefore, a deep knowledge of cellular processes must take into account cellular morpho-mechanical characterization, which should be correlated with biological data analysis. At present, cytomechanical study predominantly employs techniques that assess cellular deformability or resistance to deformation over time under controlled external forces. Atomic force microscroscopy (AFM) is the instrument of choice for this purpose, as it facilitates the investigation of different types of biological samples, from single biomolecules to entire cells and even biological tissues [24].

The assessment of the physical properties of tumor cells holds significant potential as a tool in clinical research and diagnosis [15,25]. Carcinogenesis induces a multitude of changes within cells, affecting morphology, elasticity, and adhesion properties. Therefore, quantifying these alterations and correlating them with confocal microscopy acquisitions could serve as a promising label-free indicator distinguishing tumor cells from healthy ones [26].

### 4.1. AFM Use to Investigate Structural and Morphological Changes in Cancer Cells

Normal cells typically exhibit well-structured actin filaments in their cytoskeleton, whereas tumor cells often display disorganized actin networks. Moreover, tumorigenesis can lead to changes in the mechanical properties of the tissue microenvironment. For instance, in the context of bladder cancer, tumor growth triggers the formation of muscular structures within the mucosal layer, resulting in heightened cellular rigidity across all adjacent tissue layers compared to tumoral-free samples [27]. Standardizing the characterization of these phenomena could serve to develop innovative diagnostic approaches and enhance our understanding of tumor biology.

Zhao et al. [28] investigate changes in longitudinal elasticity between human cervical squamous carcinoma cells (CaSki) and normal cervical epithelial cells (CRL2614) by performing indentation measurements at various depths in order to distinguish the contribution of the cell cortex alone from that mediated by the underlying cytoskeletal structure. CaSki cells exhibited a cortex 10 times more deformable than that of CRL2614 cells, indicative of an irregular organization of actin filaments therein.

During the metastatic process, cellular adhesion decreases, and alterations in cell shape and hardness occur [29]. Numerous studies have revealed differences in mechanical stiffness between normal and cancerous cells, as well as between primary tumor cells and metastatic cells [30].

Hayashi and Iwata [31] conducted studies on the alterations in mechanical properties of cancer cells during cervical cancer progression by using the nanoindentation method. Particularly, they measured the stiffness of non-metastatic primary cancer HeLa cells (human cervical cancer cells) and correlated these properties with those of End1/E6E7 cells (squamous epithelial cells from normal human cervix). The comparison of the obtained results indicated that cancer cells are softer than normal cells, with no significant difference in stiffness between the peripheral and central regions of the cancer cells. In contrast, normal cells (End1/E6E7) exhibited a tendency for lower stiffness in the peripheral region with respect to the central region. These experimental findings confirmed how cellular rigidity is closely connected to the organization of intracellular structures, particularly the cytoskeletal fibers, the distribution of which can undergo significant changes during carcinogenesis progression.

Kwon and colleagues [32] assessed the cellular elasticity of various cancer lines, including breast (MCF7, T47D, MDA-MB-231), cervix (HeLa, SiHa, Caski), and lung (A549, H460, H1299) lines, grown under identical conditions. They then compared the results for each type with their corresponding healthy equivalent: MCF10A, Ect1/E6E7, and WI-38 for breast, cervix, and lung, respectively. This study found that metastatic cancer cells were less soft than non-metastatic ones across all types. Specifically, breast cancer cells were 34% softer, lung cancer cells 40% softer, and cervical cancer cells 50% softer compared to their non-tumoral counterparts. Finally, the authors highlighted that elasticity is related to cell mobility, which affects metastatic potential.

**Figure 5:**
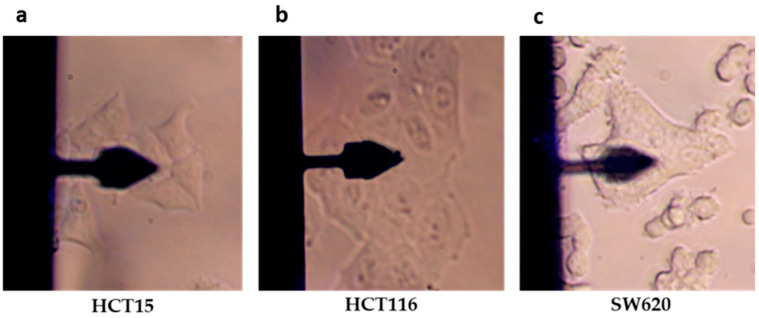
Representative images of cell types used for the quantification of their biomechanical properties using AFM (using a model PFQNM-LC-A-CAL cantilever (Bruker Gmbh, Berlin, Germany)). (**a**) HCT15, (**b**) HCT16, and (**c**) SW620 cells. Reproduced with Permission from Bras et al. [33].

In a recent study, Bras et al. [33] analyzed the viscoelastic properties of three colorectal cancer cell lines (HCT116, HCT15, and SW620) using both static and dynamic AFM methodologies (conventional FD curve acquisition and frequency sweep) to quantify cell elasticity, viscosity, and fluidity. More specifically, the approach data from FD curves acquired in FV mode were used to obtain Young’s modulus, referred to as the apparent Young’s modulus (to distinguish it from the modulus calculated from dynamic acquisitions) (Figure 5). The dynamic measurements allowed for the clear distinction of elastic and viscous contributions, yielding the storage and loss moduli dependent on the frequency.

The results showed that the apparent elastic modulus was comparable to the storage modulus. In addition, subtle differences among cell types became significant when comparing both the value of the loss tangent (the ratio of the loss modulus to the storage modulus) and the power law exponent of the storage modulus (which expressed the storage modulus as a function of frequency), both indicative of the fluidity of the cells.

This evidence could be justified by stating that the impact of the storage modulus is much greater than the loss modulus, suggesting that cellular response to loading in a force curve mainly depends on elasticity and slightly on viscosity contribution. To support this hypothesis, the authors investigated the ultrastructures of the three cell types using CLSM, simultaneously staining F-actin, tubulin, vinculin, and FAK proteins. The metastatic SW620 cells, having the highest values for the Young and storage moduli, displayed a distinct ring of cortical F-actin filaments, several FAKs, low vinculin, and the absence of filopodia. These characteristics may account for the diminished migratory activity observed in cell migration assays. On the other hand, HCT15 cells that had lower Young and storage moduli values presented high cortical tubulin, less cortical F-actin and FAK, and increased filopodia formation, most probably explaining higher migratory behavior. In the end, HCT116 cells showed high cortical F-actin expression, intermediate levels of total FAK, intermediate Young and storage moduli values compared to the other cell lines, and vast filopodia formation; this may explain the highest migratory behavior. The mechanical properties of the local environment that cancer cells adhere to could strongly influence the metastatic process and migration characteristics. In this respect, Tang et al. demonstrated in breast cancer that despite cell stiffness, the metastatic process is also influenced by the mechanical properties of the invaded organs. In this scenario, triple-negative MDA-MB-231 breast cancer cells with increased F-actin expression preferentially invaded the bone, whereas softer cells can metastasize in the brain. Accordingly, chemical inhibitors of the cytoskeletal structures (i.e., inhibitors of Cyto D, ROCK inhibitor Y27632, or myosin II inhibitor blebbistatin) can affect the expression of genes associated with the metastatic process (ADAMTS1, PTHrP, COX2, Serpin B2, LTBP1, etc.) [34].

Significant changes in cell adhesion properties during the metastatic process were revealed using AFM single-cell force spectroscopy (SCFS). In AFM-SCFS, the cell under investigation is attached to the tip of an AFM cantilever to create a cell probe, which is then manipulated vertically to perform an approach-retraction cycle towards the substrate, making possible the estimation of cell adhesion [35]. Similarly, Smolyakov et al. employed the SCFS technique to examine changes in the adhesive properties of tumor cells during invasion [36]. In this effort, they investigated the Young’s modulus and cell adhesion force of four breast cancer cell lines (SKBR3, MCF7, BT474, and MDA-MB231) with different invasiveness properties. The researchers found that invasive breast cancer cells exhibited higher adhesiveness and lower stiffness compared to non-invasive cells, which was attributed to a more significant presence of membrane tethers [36].

Moreover, the utilization of AFM in investigating the cellular morphology of leukemic white blood cells showed promising results for evaluating cell surface characteristics and detecting variations in cell surface roughness as a function of cell malignancy [37]. In a study performed by Kaul-Ghanekar et al., the researchers examined the correlation between the expression of SMAR1 protein and the roughness of cell surfaces in various stages of human breast cancer tissues. This study demonstrated the feasibility of observing the ultrastructure of tumor cell membrane surfaces and morphology and analyzing nuclear size and shape [38].

**Figure 6:**
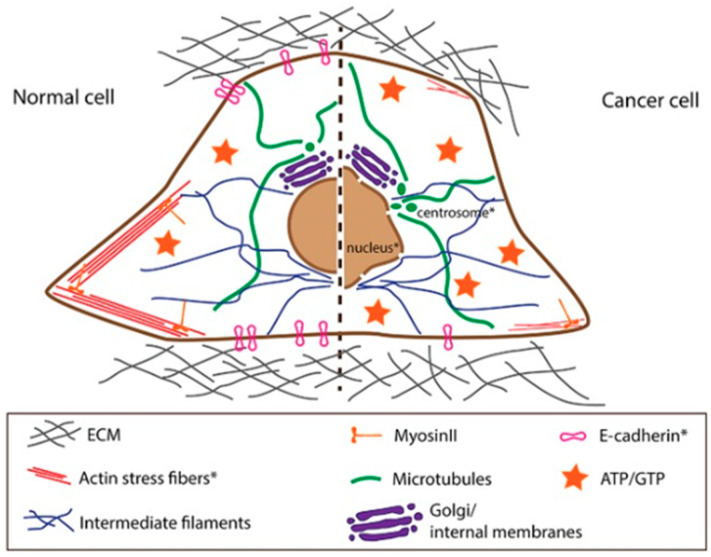
Cellular components influencing the mechanical properties of normal and cancer cells. Labeled elements (*) undergo characteristic changes during cellular transformation. The extracellular matrix (ECM), actin filaments, and intermediate filaments directly contribute to cell hardness; myosin II, E-cadherin, microtubules, energy sources (ATP/GTP), and internal membranes may also affect hardness, though their mechanisms remain unclear. Key transformations in cancer cells include (i) reorganization of the actin cytoskeleton; (ii) increased nuclear volume with deformation and perforation of the nuclear envelope; (iii) reduced density and organization of stress fibers; (iv) the presence of additional centrosomes with elongated shapes and abnormal positioning; (v) decreased E-cadherin expression. Reproduced with Permission from Alibert et al. [40].

More importantly, AFM could effectively identify significant differences in cell membrane morphology between normal and tumor cells, confirming the malignant nature of cancer cells compared to healthy cells from the same origin. Moreover, Schierbaum et al. [39] showed that confluent normal cells are stiffer and present lower fluidity than diffused normal cells, meaning they are governed by the formation of cell–cell contact. Oppositely, confluent cancer cells are not stiffer and do not show lower fluidity with respect to scattered cancer cells, and their viscoelastic properties are not dependent on cell–cell contact formation. Figure 6 illustrates the cellular elements that determine changes in mechanical properties between normal and cancer cells.

While the observed behavior of normal cells appears to be crucial for providing the mechanical stability and, therefore, the integrity of the epithelial monolayer, the dysregulation of this behavior in cancer cells might be a central aspect of early-stage cancer progression and metastasis in the epithelium (see Figure 6 and reference [40]).

### 4.2. The Role of the Tumor Microenvironment

AFM can provide insights into the pathological relationship between the metastatic process and the characteristics of the tumor microenvironment. AFM analysis of tumor extracellular matrix (ECM) micromechanics demonstrated tissue stiffness changes in relation to disease progression.

**Figure 7:**
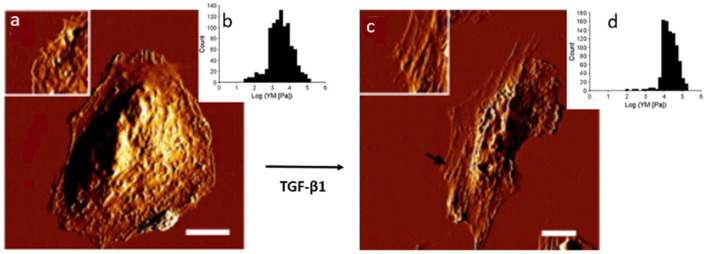
Changes in cell mechanical properties during EMT induced by TGF-β1: AFM morphology of cell before (**a**) and after (**c**) TGF-β1 treatment. The insets represent higher magnifications of the cytoskeleton organization of the cells. Cell physical properties represented by Young’s modulus bar graphs before (**b**) and after (**d**) treatment. Scale bars = 10 µM. Adapted with permission from Buckley et al. [41].

A similar combinatorial investigational approach was used by Buckley et al. to study cell mechanical properties during the epithelial-mesenchymal transition process, highlighting that tumor transformation can also be characterized by increased cell stiffness. Their study revealed that, following stimulation with transforming growth factor-β1 (TGF-β1), which was utilized to induce cellular epithelial-mesenchymal transition, the cells become more elongated (see Figure 7). This evidence was further confirmed by AFM analysis, which showed that TGF-β1 treatment resulted in an augmented number of F-actin stress fibers assembled parallel to the long axis of the cell body (see Figure 7), consequently increasing cellular stiffness [41]. More recently, Cascione and co-workers [42] used confocal laser scanning microscopy and scanning force spectroscopy to obtain a morpho-mechanical analysis on epithelial breast cancer cells (MCF-7), comparing them before and after TGF-β1 exogenous stimulation. They observed loss of cell–cell adherence due to E-cadherin expression reduction and disaggregation of actin cortical fibers in treated cells. Furthermore, the study showed that TGF-β1 induced a decrease in Young’s modulus and an alteration to nuclear morphology, suggesting the malignant progression of breast cancer cells induced by TGF-β1 exposure.

Furthermore, the same group [43] used a morpho-mechanical approach to investigate physical and structural characteristics by applying atomic force and confocal microscopy to better define the involvement of TGF-β1 in the metastatic progression process in hepatocarcinoma cells and also to evaluate the biomechanical anti-tumoral effect of LY2157299, a TGF-βR1 kinase inhibitor. In fact, the epithelial cells showed more elastic behavior after TGF-β1, suggesting enhanced migratory capability, whereas in mesenchymal cells, an opposite effect was found after LY treatment. These results envisaged the development of antimetastatic HCC therapies designed using TGF-β1 receptor inhibitors. By tuning the physical properties of the extracellular matrix (ECM) (such as stiffness), the cell mechanics behavior can be tailored. Actin filament polymerization creates a force for the protrusion of the leading edge in motile cells [44]. The created force is transferred to integrins, which pull on the ECM. A stiff ECM withstands this force in a way that the bound integrins remain immobile, whereas a soft ECM modifies under this force so that the bound integrins can also move [45]. These non-symmetric behaviors then give rise to diverse cellular answers. Most importantly, the biomechanical properties of the basement membrane (BM), an extracellular matrix (ECM) substructure of only ~100–400 nm in length, are very important for cancer progression and metastasis development. For these reasons, Hartmann and co-workers [46] reported a new protocol for quantifying the elastic modulus of the BM in murine and human lung tissue, one of the major organs involved in metastasis. They developed a methodology for calculating Young’s modulus, E, of the BM between the endothelial and epithelial cell layers, exploring the alveolar wall in lung tissues by using atomic force microscopy (AFM). Moreover, Abidine et al. [47] employed AFM-based rheology methods to investigate the viscoelastic properties of bladder cancer cells of various invasiveness on soft substrates, reporting that the rheology parameters are a marker of malignancy. The study of the micro-rheology of single cancer cells revealed that the dynamic mechanical properties are unquestionably dependent on cell type (i.e., invasiveness) and substrate stiffness. Furthermore, invasive cancer cells were found to be more mechanosensitive with respect to an augment of substrate stiffness. These findings elucidated mechanical parameters for testing biological samples in time or frequency domains, envisaging future possible therapeutic prospectives. More recently, Micalet and co-workers [48] employed an engineered 3D model, mimicking the tumor’s native biomechanical environment, and studied the changes in matrix stiffness in six patient-specific colorectal CAF populations. After 21 days of culture, AFM was used to investigate the local variation in tissue stiffness. The scope of their pioneering study was to use a 3D in vitro model of the native biophysical environment to mechanically study the changes in TME stiffness induced by patient-specific CAFs alone or in combination with colorectal cancer cells. In another publication, Stylianou et al. [49] (for the first time) reported that TGF-β induced morphological changes in CAFs, such as cell elongation and lamellipodia formation, critical factors for cell migration and invasion, which, in fact, are significantly augmented in CAFs following TGF-β treatment. The results of this study showed the effect of TGF-β on the behavior, morphology, and stiffness of CAF, giving new insights into the correlated mechanisms. The same group [50] performed in vitro experiments employing two human pancreatic cell lines in order to clarify desmoplasia, which is responsible for the stiffening of tumors, presents a strong barrier to effective drug delivery, and has been correlated with poor prognosis. Morphological and cytoskeleton characteristics, cell stiffness, and invasive properties were investigated by using optical and atomic force microscopy techniques and cell spheroid invasion assays. In a remarkable publication, Sapudom and coworkers [51] characterized the pore size and fibril diameter of collagen I networks, reconstituted by adjusting the concentration and pH value during matrix reconstitution, and showed they are prone to regulating tumor cell morphology and invasion. An analysis of the topology and mechanics of matrices was exploited using confocal laser scanning microscopy, image analysis tools, and force spectroscopy. The results show that pore size, not fibril diameter, is the main decisive factor of matrix elasticity. The authors highlighted the impact of the different hierarchical levels of ECM structures on influencing cancer cell behavior in terms of invasion and metastasis. These results envisaged the importance of using well-defined biomimetic 3D ECM matrices as in vitro models in future investigations, addressing clinically more important parameters of tumor biology. More interestingly, in a recent paper, Shen and co-workers [52] reported that tissue stiffness is higher in liver metastases than in primary colorectal tumors. Highly activated metastasis-related fibroblasts increase tissue stiffness, which increases angiogenesis and anti-angiogenic therapy resistance. Patients cured with bevacizumab showed prolonged survival when simultaneously treated with renin-angiotensin inhibitors, pointing out the importance of modulating the mechanical microenvironment for therapeutic regimens. More recently, Mahaffey et al. [53] studied the mechanical and ultrastructural characteristics of the tumor extracellular matrix (ECM) in patient-matched GBM core and rim tissues. Their results shed new light on the spatial heterogeneity of biophysical parameters in the GBM cancer microenvironment, and they analyzed the characteristics of patient prognosis, which may help in the development of strategies against resistance to therapies. Fan and co-workers [54] used polyacrylamide hydrogels and changed the physical parameters, mimicking tumor and metastatic target tissues, to explore the effect of substrate stiffness on the phenotype, growth, and chemotherapeutic solutions to ovarian cancer cells (OCCs). They revealed that increasing substrate stiffness promoted their proliferation. Notwithstanding, they showed that substrate softening increases in vimentin expression, develops elements of epithelial- mesenchymal transition (EMT), such as mesenchymal cell shape changes, and reduces E-cadherin and β-catenin expression. Growing evidence demonstrates that apart from contributing to cancer initiation and progression, EMT can promote chemotherapy resistance in ovarian cancer cells. Furthermore, we evaluated tumor response to standard chemotherapeutic drugs and found antiproliferation effects directly proportional to substrate stiffness. AFM nanomechanical studies have shown that chemosensitivity and chemoresistance are related to cellular mechanical properties. In fact, the Young’s modulus of SKOV-3 cells grown on soft substrates was found to be less than that of cells grown on stiff substrates. Their results could envisage new therapeutic targets for future anti-cancer drug designs. For further details, readers can refer to more recent review articles [55,56,57].

### 4.3. Nanomechanics and Morphological Characterization of Cellular Exosomes

AFM can also serve as a valuable tool for the in-depth analysis of cellular exosomes. Exosomes have been found to play a crucial role in tumor metastasis and intercellular communication [58,59]. A notable characteristic of cancer cells is the increased production of exosomes compared to healthy cells [60]. Exosomes can impact various factors that determine tumor malignancy, including cancer cell motility, drug resistance, and stroma formation efficiency, as well as affecting the efficacy of potential therapies [61,62]. The development of targeted exosomes can be explored as a strategy for cancer therapy, and inhibiting their function can potentially stop tumor progression and elicit a response to the treatment [63]. AFM imaging provides a clear visualization of the detailed morphology of individual exosomes, which typically requires complex pre-treatments, such as when the samples are prepared for electron and cryoelectron microscopy, potentially altering the properties of these vesicles [64]. Moreover, AFM allows for analyzing individual exosomes in liquid environments and detecting structural changes within their structure in response to different stimuli [65]. More recently, Vorselen and co-workers [66] showed how to image vesicles with minimal structural disturbance and how to analyze the data for accurate size and shape measurements. Furthermore, they described a new method for performing nanoindentation on vesicles and data analysis with mechanical models used for data interpretation. By means of different methodologies for EV morphological analysis, the generation of intracellular and extracellular vesicles was triggered by the autophagic process. Moreover, the presence of lipid raft components within secreted EVs and their physical association with the LC3-II marker might envisage the development of alternative therapies for diseases where the formation of EVs is crucial. Paul and colleagues [67] used high-resolution atomic force microscopy (AFM) and spectroscopy (AFS) techniques to detect the differences between EV colon cancer-derived and epithelial cells at the single-vesicle level, revealing that EV populations increased in the cancer cell media with respect to the normal cell EVs. This article is the first report on monitoring HA-coated EVs as a potential colon cancer biomarker that is detected in the early stages of a tumor. These results show that this is an effective approach to detecting key biomarkers from liquid biopsies, enabling the early detection of fatal colon cancer.

Recently, Ju and coworkers [68] investigated the effects of exosomes derived from hepatocellular carcinoma cells (HCC-LM3) under normoxia (N-exos) and hypoxia (H-exos) environments on the biological and physical properties of target cells. Atomic force microscopy (AFM) was used to monitor changes in the morphological and mechanical parameters of the HL-7702 cells, revealing that the cell edges became irregular and the filopodia increased upon the exosomes increasing exposition time, whereas the heights and elastic moduli of cells became smaller. This work underlines the significant effect of hypoxic tumor-derived exosomes on the invasion and the physical properties of target cells, suggesting a novel procedure for the prognosis and treatment of cancer.

Therefore, liquid biopsy-based methods to test serum proteins and extracellular vesicles as biomarkers can also help to follow brain tumors. In a proteomics-based study, Dobra and co-authors [69] aimed to identify a characteristic protein marker associated with central nervous system (CNS) cancers, showing that circulating small-sized extracellular vesicles were more useful for differentiating patient groups. This is the first quantitative investigation to compare the proteome of serum-derived small EVs with that of the original whole serum samples, showing support that EVs have a greater potential for monitoring CNS tumors compared to whole serum samples; this suggests the possible incorporation of this method into clinical practice.

**Figure 8:**
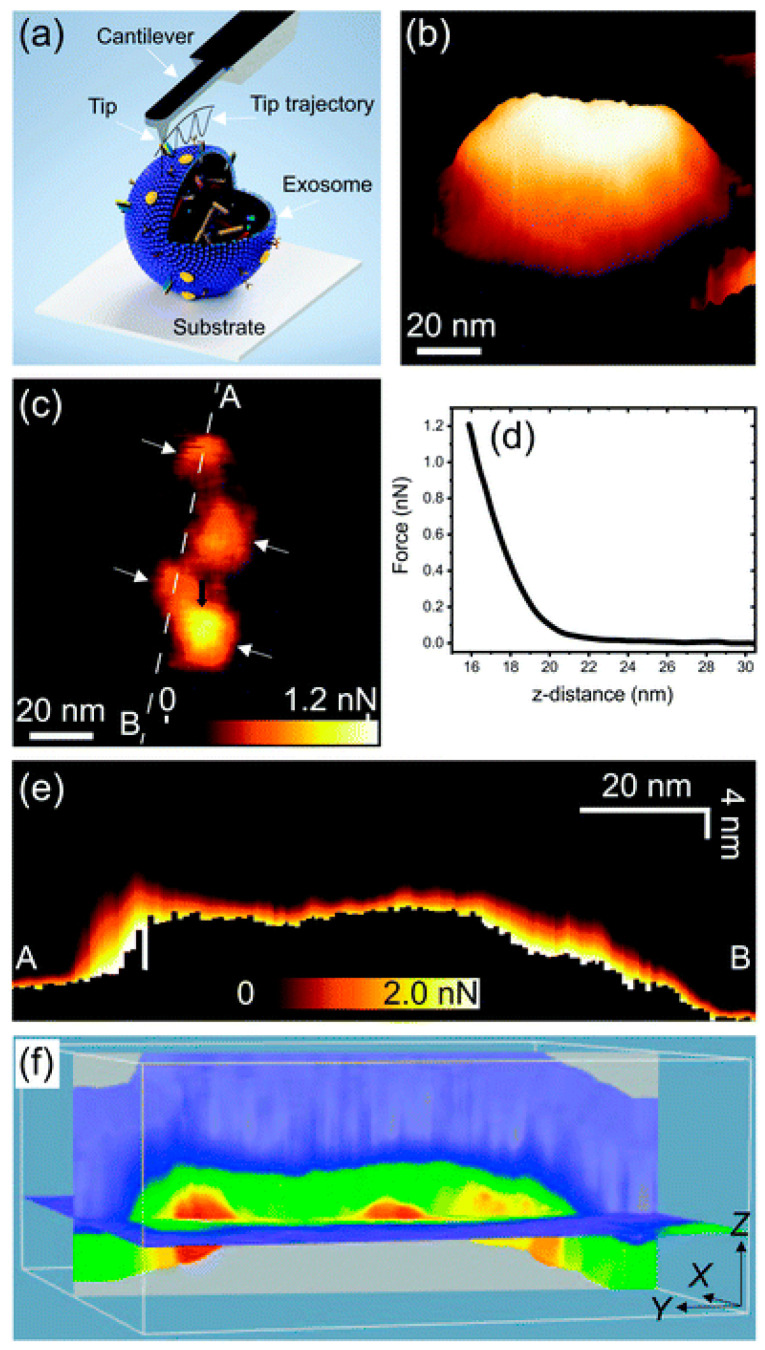
A 3D force map acquired for isolated exosomes. (**a**) Schematic representation of the 3D-AFM force mapping study. (**b**) A 3D rendering of an AFM image of an individual exosome, acquired simultaneously with the 3D-force map in panel (**c**). (**c**) A 3D-force map acquired over the exosome, shown in panel (**b**) revealing the distinction of different substructural domains (highlighted by white arrows) on the exosome surface. Four bright protruding features appear over the top of the vesicle. (**d**) A typical single force–distance curve extracted from the position marked with a black arrow in panel (**c**) indicating that there is no rupture event. (**e**) A 2D-force map profile in the zx plane obtained along the white-dashed line shown in panel (**c**). A topographic height difference of 4–5 nm can be seen between two protruding features in the 2D-force profile. (**f**) A 3D visualization of the force map by Voxler software, pointing out the surface features along the same plane as in (**e**) Two protruding features (red) can be clearly seen. The force mapping area has a dimension of 112 nm × 112 nm and is divided into grids of 256 × 256 pixels. The z-scale in panel (**b**) is 0–23.2 nm. Adapted from reference [70] with permission from the Royal Society of Chemistry.

In another study that utilized 3D-AFM, localized protrusions associated with surface membrane proteins were detected on osteosarcoma cell-derived exosomes [70]. In detail, the structural and nanomechanical properties of exosomes at high spatial resolution in physiologically relevant conditions were studied. The sub-structural details of exosomes released from different cell types were detected by force mapping. Separated local domains coming out from the exosome surfaces, related to the exosomal membrane proteins anchored on the outer surface, were investigated (see Figure 8). Exosomes derived from metastatic tumor cells carry protein content that differs from their non-metastatic counterparts, thus revealing different mechanical characteristics; this distinction between metastatic and non-metastatic malignant cell-derived exosomes evidences their application in the study of exosome biological functions and their use as diagnostic biomarkers for different types of tumors. Moreover, metastatic tumor cells release exosomes with enhanced levels of elastic fiber-associated proteins to keep their softness. In recent studies, EVs have increased expectations as a new class of diagnostics and therapeutics tools, but unfortunately, large variabilities in their isolation methods and the morpho-structural complexity of these biological nanovectors required precise biophysical characterization of single EVs. In aiming to investigate these unknown properties, several complementary techniques (such as atomic force microscopy (AFM), direct stochastic optical reconstruction microscopy (dSTORM), micro-fluidic resistive pore sizing (MRPS), or multi-angle light scattering (MALS)) have been employed by Sharma and co-workers [71]. AFM and dSTORM particle size distributions demonstrated coherent unimodal and bimodal particle size populations isolated using centrifugation and immune-affinity techniques. Furthermore, AFM imaging revealed strong differences in small EV surface nano-roughness, nanoscale morphology, and non-vesicle presence compared to various isolation methods. These results envisage the importance of the surface characteristics of single EVs, which are undetectable using standard particle sizing. By investigating the nano-structural characteristics of EVs, the comprehension of the complexity and heterogeneity of single EVs is improved, having important consequences for EV analytical research and its medical use [71].

Since EVs can modulate the biomechanical properties of target cells that have a crucial role in metastatic spreading, Senigagliesi et al. isolated and thoroughly investigated triple-negative breast cancer (TNBC)-derived small EVs. The cell stiffness, cytoskeleton/nuclear/morphology, and Yap activity rearrangements of non-metastatic breast cancer MCF7 cells upon EV treatment were assessed; the results suggest that TNBC-derived small EVs could directly induce a decrease in cell stiffness in MCF7 cells via rearrangements to the cytoskeleton, focal adhesions, and nuclear/cellular morphology, as well as augment Yap gene expression [72]

More recently, Manganelli et al. [73] studied autophagy-induced EVs in human fibrosarcoma cells using TEM and AFM. Autophagy induced the formation of large heterogeneous intracellular vesicular structures, whereas a more visible smooth cell surface, reducing the amount of plasma membrane protrusions, was also observed. Introducing a new step in the crosstalk between autophagy and the endo-lysosomal system could foster an understanding of pathogenic mechanisms, envisaging alternative therapies in diseases where the production of EVs is involved. For further details, readers can refer to review articles recently published [74,75].

### 4.4. The Role of Genes in Cancer Progression

AFM can also be used as a valuable tool for detecting tumor-associated biomarkers like the cell-free circulating DNA fragmentation index [76]. This parameter indicates changes in the genotype of the tumors [77,78], and it is particularly useful for quantifying miRNA at the single-cell level, enabling a high sensitivity in situ analysis [79]. miRNAs play a crucial role in different cellular processes, and measuring their expression levels can serve as a biomarker for cancer and its progression. Compared to AFM, conventional techniques like northern blotting and qRT-PCR are considerably less sensitive and cannot provide information on the subcellular localization of the miRNA. Significant AFM advantages can also be appreciated in comparison with enzyme-assisted fluorescence microscopy; although it can detect single molecules of miRNAs, it has limited spatial resolution [80,81]. AFM allowed for the investigation of this phenomenon in an unprecedented fashion. The mechanical forces generated by shear stress during metastasis have the capacity to trigger the expression of Yes-Associated Protein 1, a regulator of genes involved in cancer cell metastasis [82]. To investigate this phenomenon, the authors built a microchip in polydimethyl siloxane, characterized by an elastic module similar to a blood vessel. In this effort, AFM was pivotal in reproducing this kind of biomimicry. Most importantly, methylation changes at cytosine−guanine dinucleotide (CpG) sites in genes are very closely related to tumor development. Therefore, the detection and quantification of methylated DNA is pivotal for early diagnosis. In a recent report [83], an AFM-based procedure was used for DNA containing methyl-CpG at a specific site without any chemical labeling, fluorescence tagging, or amplification. The authors used AFM-tip-tethered methyl-CpG-binding proteins to detect surface-captured methylated DNA. These results envisage this force-mapping-based quantification method for future applications in the early detection of diseases correlated with methylated DNA. Chun-Hua Luo et al. [84] employed AFM to indicate that tissue stiffness was higher in isocitrate dehydrogenase (IDH) wild-type gliomas than in IDH-mutant gliomas. These results show that TIMP1 is more highly expressed and that the degree of TIMP1 promoter methylation was much lower in IDH-WT gliomas than in IDH-Mut gliomas; this promoter can also act as a prognostic biomarker for glioma. Liu and co-authors [85] showed that LRRC4 is a new MTF translocating to the nucleus as a full-length protein through endoplasmic reticulum-Golgi transport. They demonstrated that LRRC4 was bound to the enhancer element of the RAP1GAP gene for activating its transcription and inhibiting glioblastoma cell movement by influencing cell contraction and polarization. Moreover, AFM also showed that LRRC4 or RAP1GAP modified surface morphology, adhesion force, and cell stiffness.

Analyzing DNA polymorphism length and frequency in a wild-type background is difficult because of their variable and repetitive nature. Therefore, to overcome these issues, Koebley and co-workers [86] coupled synergically digital polymerase chain reaction (dPCR) and high-speed atomic force microscopy (HSAFM) to develop a fast and effective method to quantify them. Due to high throughput, single-molecule sensitivity, low costs, and a single-molecule resolution of basically any length, this coupling can be seen as a diagnostic tool for FLT3-ITDs and other length polymorphisms. On a similar pathway, Young and co-authors [87] developed a procedure to measure the mechanical properties and gene expression of single cells to generate large-linked datasets. The workflow combines AFM to measure the mechanics of individual cells with multiplexed RT-qPCR gene expression analysis on the same single cells. It was found that those genes that strongly correlated with mechanical properties were markers of extracellular matrix remodeling, epithelial-to-mesenchymal transition, cell adhesion, and cancer stemness. Furthermore, cell clustering was improved by combining mechanical and gene expression data types. This novel single-cell geno-mechanics procedure demonstrates how single-cell studies can unveil the molecular drivers affecting the biophysical processes behind metastases. Additionally, this study permitted new hypotheses concerning the association of proliferative phenotypes with a stiff mechano-type and mesenchymal phenotypes with a soft mechano-type. Finally, it will also allow researchers to envisage the molecular factors associated with the metastatic process [87].

**Figure 9:**
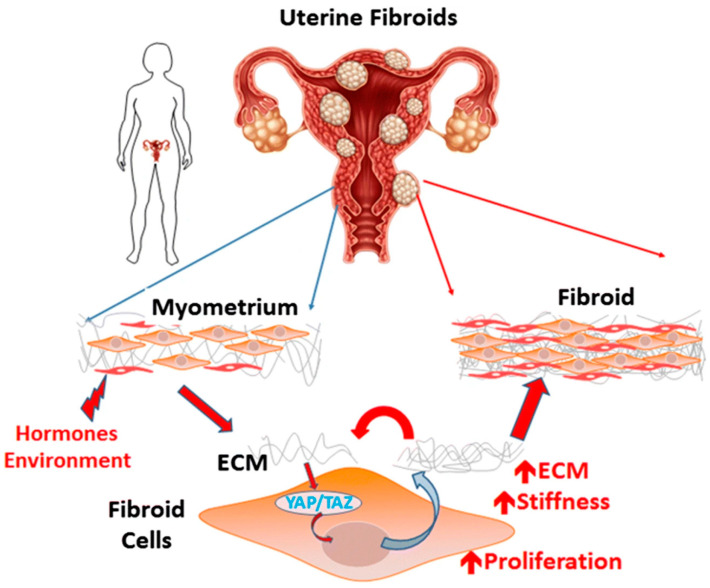
Fibroid schematic demonstrating the effects of hormones and environment (stiffness) on YAP/TAZ nuclear localization and upregulation of fibroid development. Reproduced with Permission from Purdy et al. [88].

Purdy and co-workers [88] employed AFM to monitor the in-situ stiffness of the environment activating signaling through the Hippo pathway effectors Yes-associated protein (YAP) and transcriptional coactivator with PDZ-binding domain (TAZ). They concluded that the mechanosensitive pathway can be linked to YAP/TAZ function and involves transducing fibroid growth (see Figure 9). This pathway could be a possible answer to treating long-term patients without passing through surgery [88]. For more details about the application and characterization of AFM on genes, readers are referred to recent comprehensive review articles on this field [89,90].

### 4.5. Evaluation of Drug Formulations Against Cancer Diseases

AFM can be employed to evaluate the efficiency and possible adverse effects of formulations under controlled conditions. Such studies could be useful in the design and in vitro development of emerging pharmaceutical formulations.

As aforementioned, AFM was applied to monitor the effects of specific treatments, exploiting the ability of this machine to collect data from live cells in real time and at the molecular level. The foundation of this investigation is based on the analysis of the same cell mechanics that are used to discern a healthy cell from a cancer cell. Parameters like cell adhesion to a substrate, stiffness, and elasticity can be affected by multiple treatments and cell death. In fact, whereas cell adhesion is the capacity to adhere firmly to a substrate, cell stiffness can be described via Young’s modulus. These properties are typical of a cell and can be varied, modified, and tuned by applying different treatments (such as drugs or active agents), which can also finally determine cell death. This phenomenon makes the instrument very useful in terms of better defining the working mechanism of a treatment and also gathering more information about its potential toxicity. Many therapeutics can directly or indirectly affect the ultrastructure of the cells by inducing cytoskeletal rearrangements or modulating pathways that control cytoskeleton organization. In this context, AFM was used by Yang and co-workers to investigate the clinical effects of disulfiram in nasopharyngeal carcinoma. This drug showed significant cytostatic properties on cancer cells (CNE-2Z) and slight effects on healthy counterparts (NP69-SV40T cells). In particular, disulfiram significantly decreased the roughness and increased the stiffness of CNE-2z cells because it induced the rearrangement of F-actin, Filamin-A, and α-tubulin structures, while normal cell mechanics were not affected [91].

**Figure 10:**
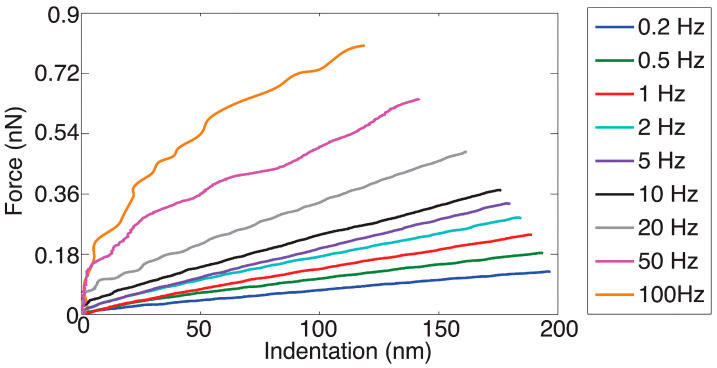
Force versus indentation curve, measured on 20 μM TPA-treated cells. Reproduced with Permission from Ren et al. [92].

In another study, PC-3 prostate cancer cell membrane viscoelasticity was evaluated via AFM measurements using Young’s modulus upon treatment with eight different chemotherapeutics (disulfiram, paclitaxel, MK-2206, Celebrex, BAY 11-7082, Totamine, 12-O-tetradecanoylphorbol-13-acetate, and valproic acid), administered at two different concentrations. All the therapeutics tested induced an increase in the Young’s modulus of the cells compared with untreated cells, and this phenomenon was attributed to actin filaments aggregation (Figure 10). Scientists also highlighted the fact that the different drugs could be grouped into two categories depending on the level of the Young’s modulus increase as a function of the exponent coefficient of the frequency modulus [92]. The dependence of the Young’s modulus on cell cytoskeleton dynamics was perfectly highlighted in the work of Huang et al., who tested the differential effects of colchicine and taxol on U937 cell elasticity. While colchicine’s working mechanism implied microtubule disassembly, taxol induced their assembly, decreasing and increasing cell strength, respectively [93].

While investigating the effects of peptides with an inhibitory effect on cysteine cathepsin activity, Rudzinska et al. demonstrated that these treatments could increase the stiffness of 786-P and A498 cancer cells and that this phenomenon was accompanied by an increase in renal and breast cancer cell differentiation and a decrease in cell motility [94].

AFM was also recently used to investigate the effects of daunorubicin (also known as daunomycin) on DNA in a cellular model [95]. This therapeutic is used to tackle different liquid tumors (lymphomas and leukemias) [96] and is characterized, like other anthracyclines, by cardiac toxicity. While daunorubicin’s working mechanism inhibits topoisomerase II activity [97], the detailed nature of the interaction between the drug and DNA is still under debate [95]. For this reason, plasmid molecules pretreated with spermine to reproduce the conditions of high-rate proliferation formed typical flower-like structures and DNA agglomerates. The AFM analysis showed clearly that daunorubicin induced double-strand breaks (DSB) and the disaggregation of this super-molecular organization, even in the absence of topoisomerase II. Actually, the use of AFM to characterize and define the number of DSB is very common in investigating the effects of chemotherapeutics on DNA. For this reason, Sofinska et al. [98] developed a new method to quantify the number and length of DNA fragments in AFM images. These parameters, in fact, were usually underestimated by the small spatial resolution of the tool. This method was based on experimental analysis obtained by treating a DNA plasmid with bleomycin, which allowed for the development of a piece of software designed to analyze and correct the systematic error of DNA fragments imaged via AFM.

Considering the large development of treatments based on monoclonal antibodies, AFM analysis became a standard technique to evaluate antibody affinity for a specific molecule. This measurement can be achieved by modifying the cantilever with an antibody and testing the binding force that results from the interaction with its ligand. This approach was used by Kim et al. to investigate antibody affinity towards EGFR in A431 cancer cells and the density of this biomarker on the cell surface [99]. A similar approach was used to test the affinity of human luteinizing hormone-releasing hormone (LHRH) peptides for their receptors and of antibodies specific to the EphA2 receptor on breast cancer cells [100]. In MDA-MB-231 breast cancer cells, the high content of overexpressed LHRH or EphA2 receptors showed a very strong affinity for LHRH peptides or anti-EphA2 antibodies. These data were compared with the same analysis performed on healthy Hs578Bst breast cells, which exhibited only limited interactions with these ligands. In this case, AFM was useful for estimating the specificity of targeting molecules [100].

In many cases, AFM was used to investigate the relation between cell mechanics, biology, phenotype, and treatment. For example, Wang et al. investigated curcumin for its ability to decrease the expression of the surface biomarker CD44 of HepG2 cells. In parallel, they investigated cell roughness in response to the treatment, and AFM analysis allowed for correlating the increase of this parameter in association with curcumin treatment and a consequent CD44 expression decrease on the cell surface [101]

AFM was also used to investigate the effects of antibody-based treatment on cell mechanics. Cetuximab, an antibody specific for the epidermal growth factor (EGF) receptor, was administered to A549 lung cancer cells in comparison with the natural ligand EGF. While cell stiffness decreased after EGF treatment (that induces cell proliferation), cetuximab could increase this parameter significantly, demonstrating a direct relationship between the treatment and the cell’s mechanical properties [102].

With the same aim, Li et al. used AFM to define the working mechanism of different therapeutics, offering a new approach to evaluate drug synergistic effects. In particular, the morphology and mechanical properties of lymphoma cells were evaluated upon treatment with rituximab (a targeted monoclonal antibody against CD20), cisplatin, and cytarabine (small molecule therapeutics). The scientists identified a precise cell mechanics profile induced by each drug, alone or in combination, developing an analytic approach that might improve the prediction efficacy of drug combinations before their clinical testing [103].

Similarly, in another recent study [104], the effects of cisplatin, docetaxel, and zinc (II) were tested on the stiffness of prostate cancer cell lines defined by a different malignant phenotype. The analysis showed that the stiffness of the untreated cells was not always inversely proportional to their metastatic potential, as expected, but after treatment with docetaxel and cisplatin, cell stiffness increased in all the samples analyzed. These data directly correlate with the expression of Cav-1, which was hypothesized to represent a biomarker of cell stiffness together with cell wet/dry mass ratio.

In another study, colon cancer cells (CaCO-2) were challenged with mevastatin and compared with untreated cells and healthy colon cells (CCD-18Co) [105]. Compared to healthy cells, CaCO-2 showed a reduced Young’s modulus and adhesion force, and they showed an increased indentation. This method is used to evaluate the hardness of materials. This implies making an indent on the material surface and measuring the residual plastic impression in the specimen as a function of the applied load, thereby measuring the area of contact of the indenter with the specimen. As with many other statins, mevastatin treatment induced changes in cell mechanics, reverting these parameters in CaCO-2, indicating values similar to healthy cells [105]

Nonetheless, AFM is becoming one of the gold standard procedures for characterizing the shape and the stiffness of nanotherapeutics. This technique, in fact, has numerous applications in material science, and in the context of nanoparticles, it can confirm particle size, shape, mechanical properties, and surface functionalization. AFM is also useful for understanding the changes in cell mechanics occurring in response to particle internalization, and in particular, at the interface between cell membrane and nanoparticles.

**Figure 11:**
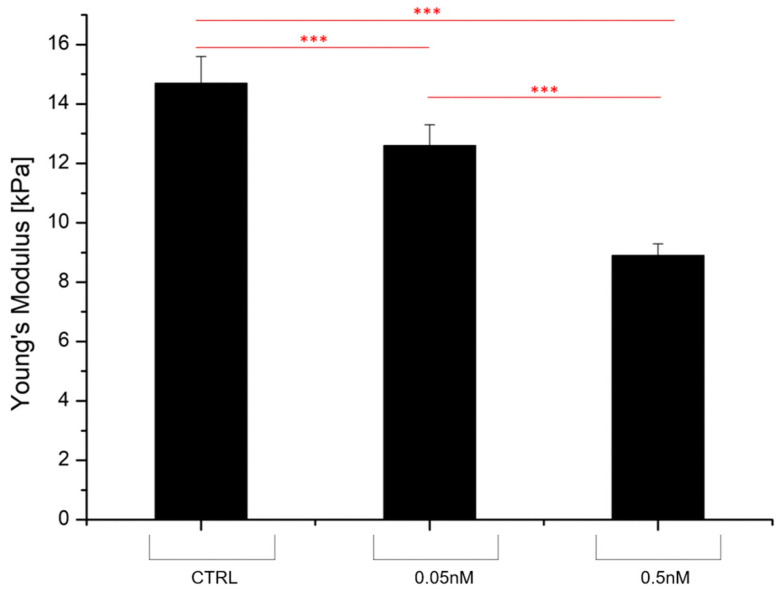
Young’s modulus values [kPa], calculated from the cytoplasmic area of MCF-7 after exposure to 0.05 nM and 0.5 nM of AgNPs for 48 h. Results were statistically significant for *p* < 0.05 (<0.005 ***). Reproduced with Permission from De Matteis et al. [106].

In this respect, in a pioneering article, De Matteis and co-workers [106] examined the toxicity of citrate-capped AgNPs on cortical actin mitochondria and lysosomes on MCF-7 epithelial breast cancer cells. Viability, oxidative stress, mitochondria membrane potential alteration, and apoptosis activation were assessed upon NP uptake. The quali-quantitative morphological alterations of cortical F-actin and organelles were quantified by confocal microscopy coupled with biomechanical analysis by means of atomic force microscopy (see Figure 11). Similarly, Cascione et al. [107] showed that exposure to Titania nanoparticles produces alterations to cellular membrane elasticity, owing to actin proteins cytoskeleton rearrangement in nuclear as well as in cytoplasmic regions. The authors have evidenced strong cell membrane mechanical property modification induced by nanoparticle interactions [107].

**Figure 12:**
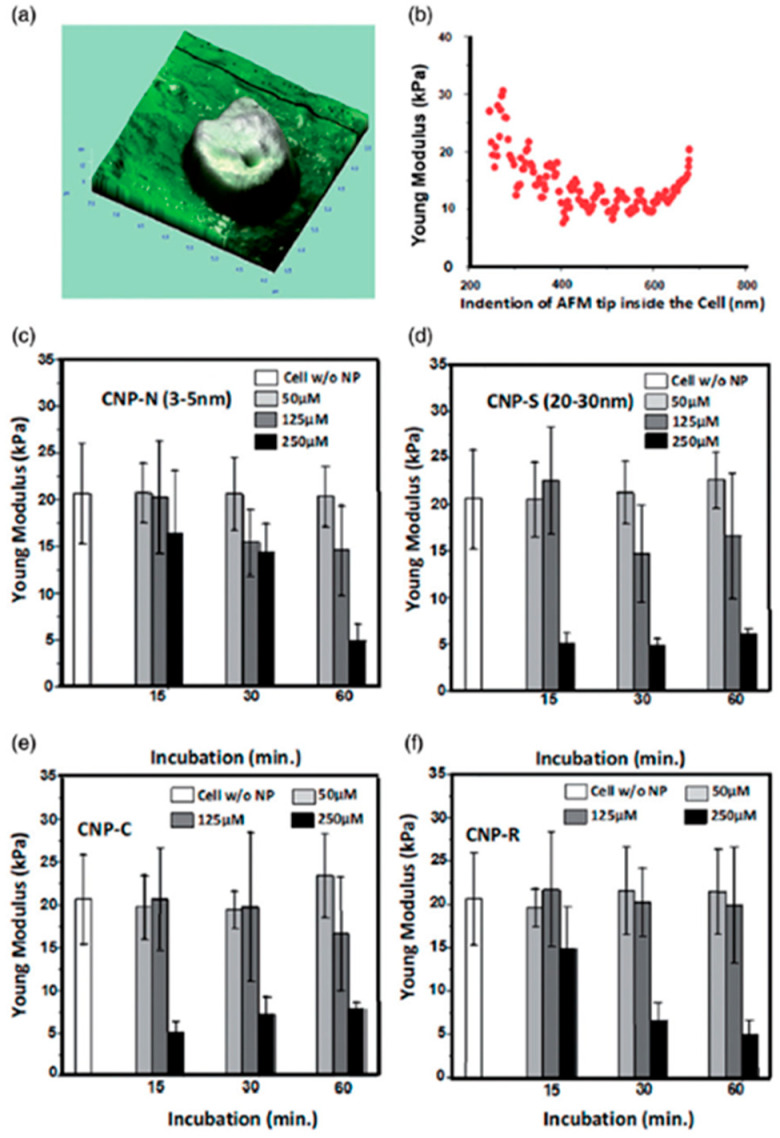
The Young’s modulus of cells treated with cerium oxide nanoparticles of different sizes. (**a**) Image of cell indentation scanned by AFM. (**b**) The Young’s modulus of the cells versus the indentation of the AFM tip inside the cell, each red points represent single indentation inside the cell. (**c**) Young’s modulus of cerium oxide nanoparticles (3–5 nm) cells versus incubation time at different particle concentrations and time period of incubation. (**d**) Cerium oxide (20–30 nm) nanospheres cells (Young’s modulus data). (**e**) Cerium oxide nanocubes cells (Young’s modulus data) and (**f**) cerium oxide nanorods cells (Young’s modulus data), derived after treating with different concentrations and time periods of incubation. Reproduced with Permission from Singh et al. [108].

In the same context, it was also shown that treating squamous cell carcinoma cells CCL30 with cerium nanoparticles could decrease the cell membrane Young’s modulus in a concentration-dependent way, and, at high concentrations, this phenomenon was also directly related to particle size [108] (see Figure 12).

Lara-Cruz et al. demonstrated that the internalization of gold nanoparticles could increase the roughness of the cell membrane of breast cancer cells, in particular when the treatment was combined with estradiol; this phenomenon could, in turn, increase the carrier cellular uptake [109].

Recently, AFM was used to demonstrate the fact that nanomaterials can increase cancer intercellular and cell-matrix adhesion, inhibiting their motility and potentially decreasing their metastatic behavior in the MFC-7 cell line. In particular, it was shown that clay nanoparticles (comprising Na-montmorillonite, Hectorite, and Palygorskite particles) can increase cell adhesion with other cells and with fibronectin by bridging these biological elements through electrostatic and hydrophobic bonds [110]. This phenomenon was demonstrated by developing a particular setting of AFM focused on measuring the maximum forces necessary to separate the cells from the substrate. The scratch-induced wound healing assay confirmed that the nanoparticles could inhibit cell motility, opening a new scenario in the development of technologies designed to target cell metastases and migration. Miletić et al. applied AFM together with Raman spectroscopy to follow the molecular and morphological changes occurring during the interaction of HeLa cells with dextran-coated and uncoated cerium dioxide (CeO2) nanoparticles [111]. The AFM results demonstrated that the coating could affect cell roughness and height, providing an accurate analysis that is impossible to achieve with classic label-free methods.

In another study, Ali et al. [112] developed gold nanoparticles to target HEY A8 ovarian cancer cell nuclei. This phenomenon can increase the stiffness of this organelle by increasing the expression of Laminin A/C and eventually negatively impact cell migration [113]. In this work, AFM was used to show that gold nanoparticles were efficient in increasing nuclear stiffness. AFM was recently used to understand the dynamics governing the interaction between leukemic cells and titanium nanoparticles. The investigation of this phenomenon is important because these particles can be used for photodynamic therapy ex vivo, and leukemic cells preferentially adhere to these carriers. The analysis demonstrated that during the initial stages of cell adhesion, a cyclic pattern of expansion and contraction occurred, eventually transitioning into a steady state [114]. In addition to the therapeutic standpoint, these data are important because they allowed for the in-depth study of the amoeboid movements of cancer non-adherent cells. Adhesion loss is also a sign of cell death, and AFM can investigate this phenomenon precisely. Here, the cells were seeded on the AFM cantilever, affecting its deflection ability and resonance frequency. Under exposure to toxic chemicals and nanoparticles, changes in these parameters could be measured as a function of cell detachment (and death). Compared to traditional cytotoxicity methods, this approach is label-free and allows for real-time investigation of cell toxicity. This method could have a big impact on the miniaturized analysis of single cells or primary cell cultures [115].

An interesting perspective development of AFM, referred to as magnetic force microscopy (MFM) [116], is very useful in measuring the magnetic properties of biological samples. The system relies on non-contact measurement between the sample exerting the magnetic field and the magnetized AFM tip. This setup allows for imaging the magnetic domain at nanoscale resolution and single-molecule resolution. MFM is currently interrogated to unveil the effect of the magnetic field on biological samples, including the biological molecules that can sense this force; the results are particularly useful in synthesizing and characterizing magnetic nanoparticles that can eventually be used for thermo-ablation [117].

AFM was also used to investigate treatment side effects, such as its application in investigating morphological changes in the red blood cells of patients receiving radiotherapy [118]. Irradiation can induce a dose-dependent decrease in the Young’s modulus of the red blood cells, probably by affecting the cytoskeleton organization and morphological abnormalities comprising nanoparticle formation on the cell membrane [119]. However, the scientists concluded that radiotherapy might not be the only reason for this observation since the increased generation of extracellular vesicles in cancer patients might be the primary cause of this phenomenon [119]. Furthermore, Ma and co-workers [120] exploited the dynamic mechanical properties of single cells under various drug conditions, designing two mathematical approaches for cell physiology. The cellular mechanical properties upon drug activation were shown to increase according to time and saturation, and this was mathematically explained using a linear time-invariant dynamical model. Actually, the cytoskeleton structure of a cell is much denser, and there is a linear correlation between its cytoskeleton density and the single components of its Young’s modulus and its multiple viscoelasticity properties. Therefore, the physiological state of a cell in terms of its cytoskeleton density can be obtained using a linear regression model through its multiple viscoelasticity properties or by knowing its Young’s modulus. These studies pointed out that the proposed approaches can mathematically investigate the physiological state of cells and quantitatively reveal the drug efficacy on cells by relating it with their dynamic mechanical properties at the single-cell level [120].

Notwithstanding, as shown previously, the integration of AFM with advanced optical and fluorescence microscopy techniques has revolutionized the study of cellular mechanics and biochemical processes. For this reason, compared to other notable reviews on this topic, we have placed particular emphasis on the integration of these technologies, which serve as powerful tools for investigating complex cellular behaviors. These systems enable deeper insights into critical processes such as membrane dynamics, protein interactions, and intracellular signaling. By combining AFM with advanced imaging methods (i.e., confocal microscopy, FLIM, FRAP, FLIM, and STED), researchers can simultaneously capture high-resolution structural details, monitor dynamic processes, and correlate mechanical responses with molecular interactions [121,122].

Finally, the potential integration of AFM and its multiple setups with holotomographic microscopy (HTM) is worth mentioning. HTM is an advanced, label-free optical technique for the 3D imaging of live cells and nanoparticles. Measuring refractive index differences provides nanoscale resolution, quantitative data (e.g., protein concentration and dry mass), and 3D reconstructions without using dyes or phototoxicity. By using low-energy lasers, HTM enables a fast, non-invasive analysis of cellular structures and dynamic processes, making it a powerful tool for biomedical research. The integration of AFM with HTM provides a complementary and robust approach to cancer research by combining the precise surface imaging and nanomechanical profiling of AFM with the label-free, 3D visualization of internal cellular structures provided by HTM. This integration allows for the non-invasive, real-time analysis of cancer cells, connecting mechanical traits like elasticity and adhesion to internal features such as nuclear morphology and chromatin organization. By capturing nanomechanical fingerprints for early cancer detection and monitoring cellular changes during drug treatments, this hybrid approach offers valuable insights into cancer progression, diagnosis, and therapeutic evaluation, establishing AFM and HTM as advanced critical tools in oncology research [123].

## 5. Conclusions and Future Perspectives

In this review, current AFM practice is revealed to be a versatile tool to assess not only morpho-structural surface properties in biological and bio-medical samples but also to investigate their nanometer-scale mechanical characteristics and obtain important insights about their mechanisms and molecular pathways. Moreover, AFM may represent a strategic technique to investigate the physical properties and functions of the immune system, such as mechano-sensing, -signaling, and -transduction; this could help researchers unveil and understand the mechanistic role of immunotherapy for future neuronal diseases and cancer treatments. Some of the main limitations of biological sample AFM imaging are the requirement to be firmly anchored to the substrate and the slow acquisition speed of the AFM images; the latter has already been overcome by using novel small cantilevers and high-speed AFM systems that are able to follow very fast biological processes by acquiring several frames per seconds without losing resolution or information. Nevertheless, one may also expect specific improvements in electronics, AFM mechanics, and even new bio-specific techniques in the future, which may open future fields of investigation. On the other hand, the development of miniaturized, automated, user-independent, and easy-to-use AFM systems could also increase the number of possible access points to AFM in clinical uses. By improving sensitivity and thermal stability, future developments in AFM systems, coupled with the standardization of the imaging and force mapping acquisitions, analysis, and quantification methods, could pave the way to finally reveal hidden cancer cell tissue properties and complex pathways. One may also envisage that the combined use of super-resolution optical microscopy and AFM could become a new futuristic weapon for physicians to assess an early diagnosis and improve the fight and/or cures against serious diseases like cancer. In the context of tumor treatment advances, AFM might be pivotal to opening new avenues of pharmacological investigation and following the effect of novel and established therapeutics in real time.
